# Design, Synthesis, and Anticancer Screening for Repurposed Pyrazolo[3,4-d]pyrimidine Derivatives on Four Mammalian Cancer Cell Lines

**DOI:** 10.3390/molecules26102961

**Published:** 2021-05-16

**Authors:** Eman M. Othman, Amany A. Bekhit, Mohamed A. Anany, Thomas Dandekar, Hanan M. Ragab, Ahmed Wahid

**Affiliations:** 1 Department of Bioinformatics, Biocenter, University of Wuerzburg, Am Hubland, 97074 Würzburg, Germany; eman@toxi.uni-wuerzburg.de (E.M.O.); dandekar@biozentrum.uni-wuerzburg.de (T.D.); 2 Department of Biochemistry, Faculty of Pharmacy, Minia University, Minia 61519, Egypt; amany_bkeet@mu.edu.eg; 3 Division of Molecular Internal Medicine, Department of Internal Medicine II, University Hospital Wuerzburg, 97080 Würzburg, Germany; mohamed_M@klinik.uni-wuerzburg.de; 4 Division of Genetic Engineering and Biotechnology, Department of Microbial Biotechnology, National Research Centre, Giza 12622, Egypt; 5 Department of Pharmaceutical Chemistry, Faculty of Pharmacy, Alexandria University, Alexandria 21500, Egypt; 6 Department of Pharmaceutical Biochemistry, Faculty of Pharmacy, Alexandria University, Alexandria 21500, Egypt

**Keywords:** pyrazolo[3,4-d]pyrimidine, anticancer activity, apoptosis, Ki67

## Abstract

The present study reports the synthesis of new purine bioisosteres comprising a pyrazolo[3,4-d]pyrimidine scaffold linked to mono-, di-, and trimethoxy benzylidene moieties through hydrazine linkages. First, in silico docking experiments of the synthesized compounds against Bax, Bcl-2, Caspase-3, Ki67, p21, and p53 were performed in a trial to rationalize the observed cytotoxic activity for the tested compounds. The anticancer activity of these compounds was evaluated in vitro against Caco-2, A549, HT1080, and Hela cell lines. Results revealed that two (**5** and **7**) of the three synthesized compounds (**5**, **6**, and **7**) showed high cytotoxic activity against all tested cell lines with IC_50_ values in the micro molar concentration. Our in vitro results show that there is no significant apoptotic effect for the treatment with the experimental compounds on the viability of cells against A549 cells. Ki67 expression was found to decrease significantly following the treatment of cells with the most promising candidate: drug **7**. The overall results indicate that these pyrazolopyrimidine derivatives possess anticancer activity at varying doses. The suggested mechanism of action involves the inhibition of the proliferation of cancer cells.

## 1. Introduction

Cancer is considered a serious health hazard problem. Indeed, cancer is ranked the second cause of recorded deaths in the world. More than 9 million deaths were recorded due to cancer in 2018 [1].

About 68,800 novel cancer cases are recorded in Egypt every year. Liver cancer is prevalent in Egypt. This is most likely due to the spread of the hepatitis C virus among the Egyptian population. Other types of cancer that exist in Egypt include bladder, non-Hodgkin lymphoma, leukaemia, lung, and prostate cancers [2,3].

The cost of anticancer drugs is increasingly being recognized as a global problem. The price of cancer treatment in Egypt is exorbitant and may delay both diagnosis and treatment. A study was performed in order to conduct cost analysis to assess expenditures for acute myeloid leukaemia (AML) treatment in Egypt from 1999 to 2002. In the study, the median total cost of therapy per patient was $16,701 (or 33,158 LE, comparatively high for the average household therapy) [4].

**Figure 1:**
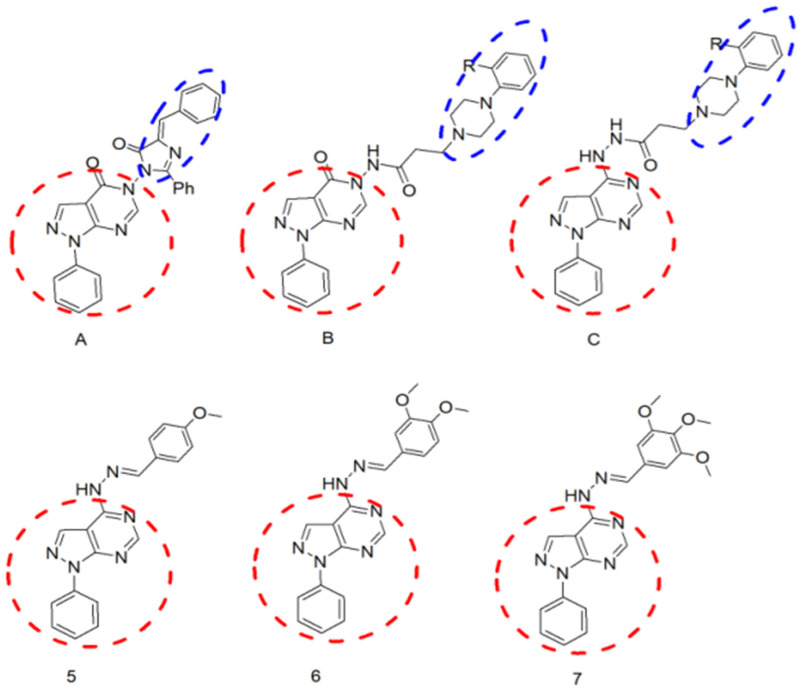
Chemical structure of previously reported compounds (**A**–**C**) [10] as a scaffold for the design of the newly synthesized compounds (**5**–**7**).

Purines are an important scaffold for the design of potent antitumour agents. They were modified using the bioisostere pyrazolo [3,4-d] pyrimidine as the central nucleus of the synthesized compounds [5]. Ghorab et al. synthesized a group of pyrazolo[3,4-d]pyrimidines substituted with a diazole moiety bearing different aromatic nuclei (compounds of the general formula A; Figure 1) [6]. Some of the synthesized compounds showed moderate cytotoxic activity. A 2-phenylaminopyrimidine called imatinib is specifically used for the treatment of gastrointestinal stromal tumours and chronic myelogenous leukaemia (CML) [7]. In addition, ibrutinib is a pyrazolopyrimidine-based kinase inhibitor, which has been studied for the treatment of B-cell cancers [8]. Pyrazolopyrimidine modulates many signal pathways that regulate cell proliferation, growth, and apoptosis [9].

Furthermore, the abovementioned findings led to the design and synthesis of a group of compounds that comprise the pyrazolo[3,4-d]pyrimidine scaffold linked to a piperazine moiety, bearing different aromatic nuclei through different amide linkages (compounds of the general formulas B and C; Figure 1), as well as to tests of their anticancer activity [10]. Finally, further screening of literature revealed that the introduction of a 3,4-dimethoxybenzylidene moiety to the hydrazine group at the 4-position of the abovementioned nucleus resulted in a new compound (**6**; Figure 1): this was capable of inhibiting BCR-ABL tyrosine kinase, an enzyme highly implicated in cell proliferation leading to the development of tumours. In addition, the inhibitors may bind to the ATP-binding pocket and restrain the ATP-binding capacities of other kinases, which may diminish their proliferative effects [11]. However, the study tested the effect of such an action on treating type 2 diabetes mellitus (T2DM).

Thus, it was the goal of this study to design and synthesize a group of compounds that comprise the N-aryl pyrazolo[3,4-d]pyrimidine scaffold having different arylidenehydrazino substitution at the 3-, 4- and 5-positions (compounds **5**–**7**) and to test their anticancer activity. Synthesized compounds are expected to retard the progression of malignancies. The substitution pattern of the synthesized compounds was carefully selected so as to confer different electronic and lipophilic properties to molecules.

Accordingly, we investigated if the newly synthesized pyrazolo[3,4-d]pyrimidine derivatives could significantly improve cancer therapy. We also identified the primary mechanism of action of the new drugs.

Furthermore, in silico docking experiments of the synthesized compounds against Bax, Bcl-2, Caspase-3, Ki67, P21, and P53 were executed, aiming at providing insights into the observed cytotoxic and antiproliferative activities for the tested compounds.

## 2. Results

### 2.1. Chemistry of the Compounds under Investigation in the Current Study

**Figure 2:**
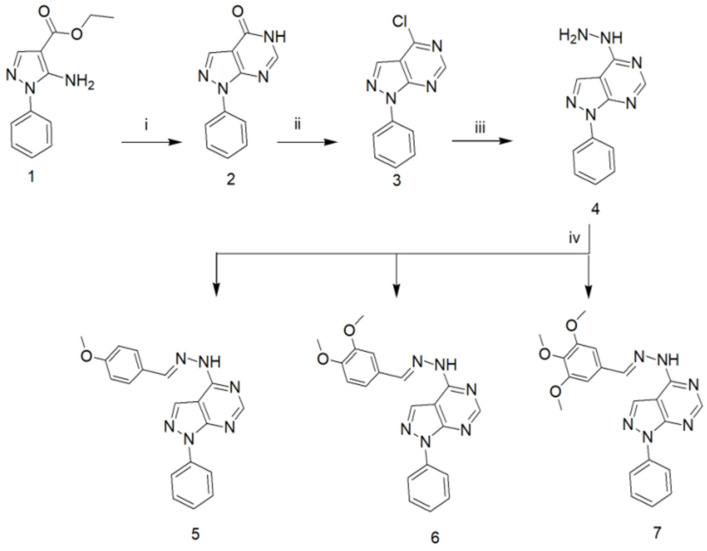
Synthetic pathway for the synthesis of the target compounds **5**–**7**; reagents and reaction conditions: i: HCONH_2_, reflux, 10 h; ii: POCl_3_, reflux, 4 h; iii: NH_2_NH_2_, EtOH, reflux, 6 h; iv: ArCHO, EtOH, reflux, 3 h.

The synthetic strategies employed for the synthesis of the target compounds in the current study are illustrated in (Figure 2). Compounds **1**–**4** were synthesized in accordance with the methods described in the literature [12,13]. Compounds **5**–**7** were obtained through condensation of the hydrazine compound (**4**) with the appropriate aromatic aldehyde. The ^1^H-NMR spectra lacked the two D_2_O-exchangeable protons corresponding to the NH_2_ and revealed instead the appearance of a benzylidene CH proton at its expected chemical shift. Furthermore, the spectra indicated the appearance of additional aromatic protons at δ 7–8 ppm corresponding to the aromatic aldehyde aromatic protons together with the methoxy protons at their expected chemical shifts. Finally, ^13^C-NMR of the synthesized derivatives indicated the appearance of a signal corresponding to the benzylidene carbons in addition to the methoxy carbons at their expected chemical shift.

### 2.2. In Vivo Toxicity Evaluation

Before the assessment of the anticancer activity of the compounds, in the present study, we examined their toxicity in vivo on normal tissues. The treated rats with 20 mg/kg/day did not show significant toxicity in comparison to the control group (Table 1). The results indicate the possible future application of the studied compounds in vivo with lower side effects.

### 2.3. Docking Analysis

The docking simulation for the ligand was carried out using molecular operating environment (MOE) software supplied by the Chemical Computing Group, Inc., Montreal, QC, Canada [14]. Six different protein targets were examined for the binding site; the selected proteins are the most common proteins in the cytotoxicity process, resembling different mechanisms for cytotoxicity. Studying the ligand interactions of all six proteins, it was found that tightly bound conserved water molecules are of importance in the protein–ligand complex binding sites of three of them (Caspase-3, Bax, and P53). Accordingly, their docking simulations were done without the removal of the water chains.

#### 2.3.1. Docking of the Test Compounds (**5**–**7**) in the Binding Site of Ki67 Protein

Docking studies were carried out using the enzyme parameters obtained from examining the structure of Ki67 (Protein Data Bank (PDB) ID: 2aff) [15].

The binding modes for the test compounds synthesized in our study (**5**–**7**) were analysed, and the following results were obtained.

**Figure 3:**
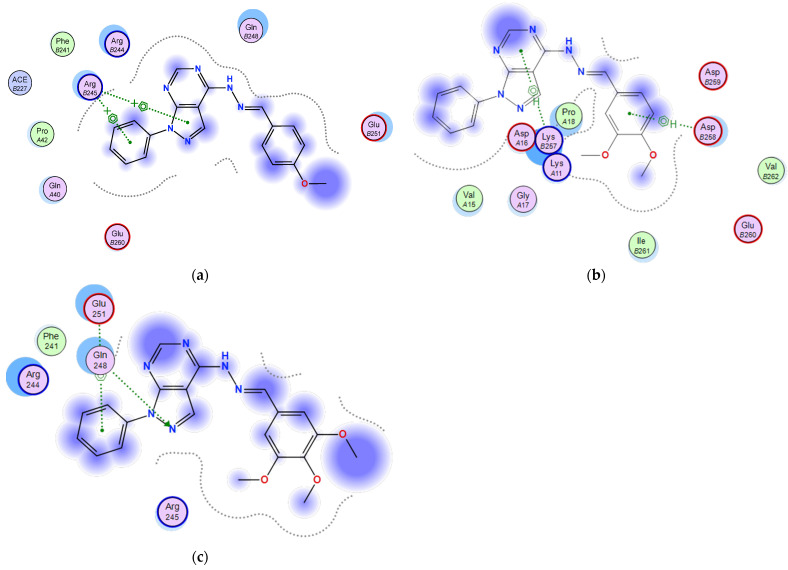
Two-dimensional binding mode of the test compounds (**a**) **6**, (**b**) **5**, and (**c**) **7** in the binding site of Ki67 protein (PDB ID: 2aff) using MOE software.

In the case of the binding mode of compound **5** (Figure 3a), the results revealed a binding score equal to (−4.5039). The X-ray crystal structure revealed the presence of two arene–cation bonds between Arg245 and the phenyl and pyrazole rings, in addition to various hydrophobic interactions with other amino acids.

The binding mode of **6** (Figure 3b) showed a binding score equal to (−4.4699). The X-ray crystal structure revealed two arene–H bonds, one between the pyrimidine ring and Lys257 and the second between the phenyl and Asp258, in addition to hydrophobic interactions with several amino acids.

Figure 3c shows the binding mode of **7**, indicating its superior binding score of (−6.5176) relative to **5** and **6** (−4.5039 and −4.4699, respectively). The X-ray crystal structure revealed hydrogen bonding between the pyrazole unsubstituted-N and Gln248 and an arene–H bonding between the N-phenyl and Glu251, in addition to the hydrophobic interactions with several other amino acids.

#### 2.3.2. Docking of the Test Compounds (**5**–**7**) in the Binding Site of Caspase-3 Protein

The docking study was carried out using the enzyme parameters obtained from Protein Data Bank of the structure of caspase-3, having the PDB ID: 6bdv [16].

Running the binding simulation for the tested compounds (**5**–**7**) gave the following results

**Figure 4:**
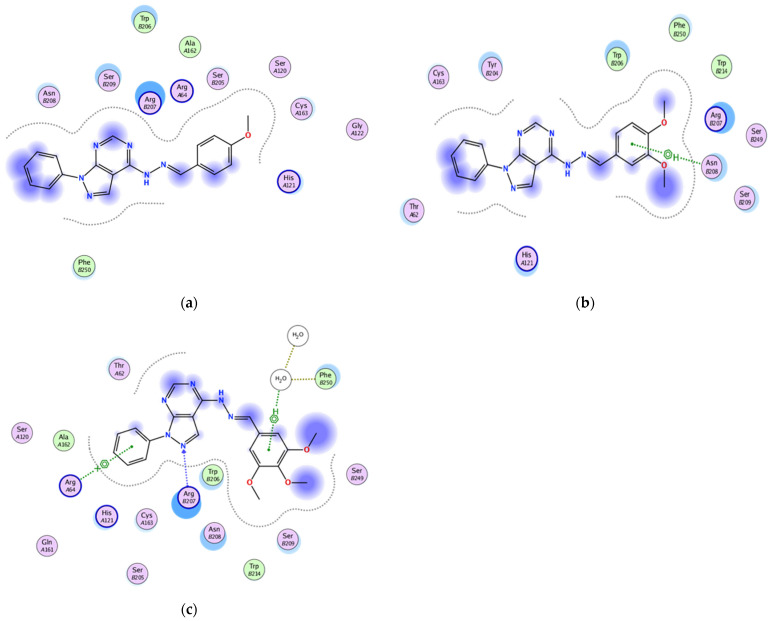
Two-dimensional binding mode of the test compounds (**a**) **6**, (**b**) **5**, and (**c**) **7** in the binding site of caspase-3 protein (PDB ID: 6bdv) using MOE software.

In the case of **5** binding mode (Figure 4a), the results revealed a binding score equal to (−5.838). The X-ray crystal structure revealed only various hydrophobic interactions with other different amino acids.

The binding mode of **6** (Figure 4b) showed a binding score equal to (−5.0599). The X-ray crystal structure revealed arene–H binding between the phenyl ring and Asn208, in addition to hydrophobic interactions with several amino acids. Figure 4c shows the binding mode of **7**, indicating a binding score of (−5.7119). Furthermore, it indicates the presence of an arene–cation bonding between Arg64 and the pyrazolo-phenyl ring together with a hydrogen bonding between the pyrazolo-N and Arg207. Furthermore, two water molecules were involved in a bond between the phenyl ring and Phe250, in addition to the hydrophobic interactions with several other amino acids.

#### 2.3.3. Docking of the Test Compounds (**5**–**7**) in the Binding Site of p53 Protein

This docking study was carried out using the enzyme parameters obtained from the Protein Data Bank structure of p53 (PDB ID: 5mct) [17].

To predict the binding modes for the test compounds (**5**–**7**), docking simulations were performed, and the following results were obtained.

**Figure 5:**
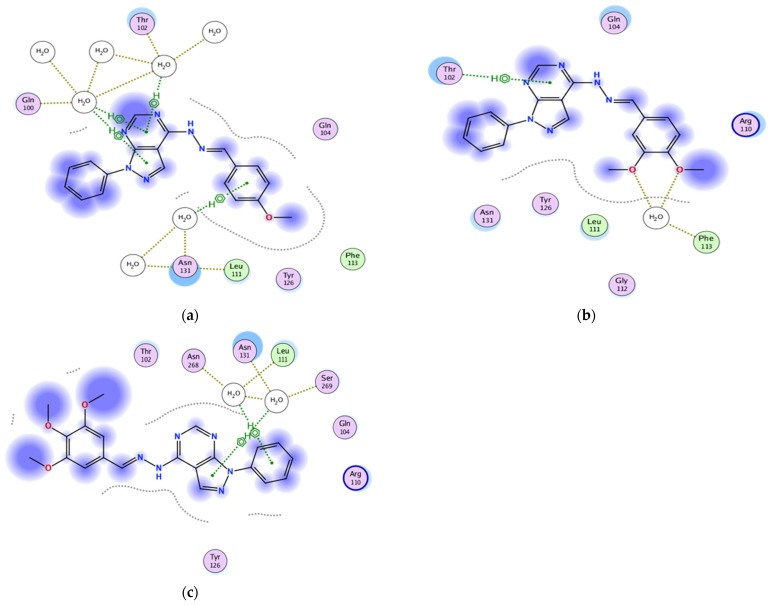
Two-dimensional binding mode of the test compounds (**a**) **6**, (**b**) **5**, and (**c**) **7** in the binding site of P53 protein (PDB ID: 5mct) using MOE software.

In the case of the binding mode of compound **5** (Figure 5a), the results revealed a binding score equal to (−5.2404). The X-ray crystal structure revealed that the compound forms four arene–H bonds with the protein, the first between the pyrazole ring and Gly112 and the second between the pyrimidine ring and Arg110, in addition to two bonds between the N-phenyl and Hist115 and Phe113. Furthermore, various hydrophobic interactions with other amino acids were observed.

The binding mode of **6** (Figure 5b) showed a binding score equal to (−4.9425). The X-ray crystal structure revealed that the compound forms two arene–H bonds with the protein, the first between the pyrimidine ring and Arg110 and the second between the N-phenyl and Phe113, in addition to hydrophobic interactions with several amino acids.

Figure 5c shows the binding mode of **7**, indicating a superior binding score of (−5.3192) relative to **5** and **6** (−5.2404 and −4.9425, respectively). Furthermore, it showed hydrogen bonding between the pyrimidine-N and Gln104 and an arene–H bonding between the N-phenyl and Tyr126, in addition to the hydrophobic interactions with several other amino acids.

#### 2.3.4. Docking of the Test Compounds (**5**–**7**) in the Binding Site of the Bax Protein

A docking study was carried out using the enzyme parameters obtained from the structure of Bax (PDB ID: 6eb6) [18].

Performing the binding modes for the test compounds (**5**–**7**) revealed to the following

**Figure 6:**
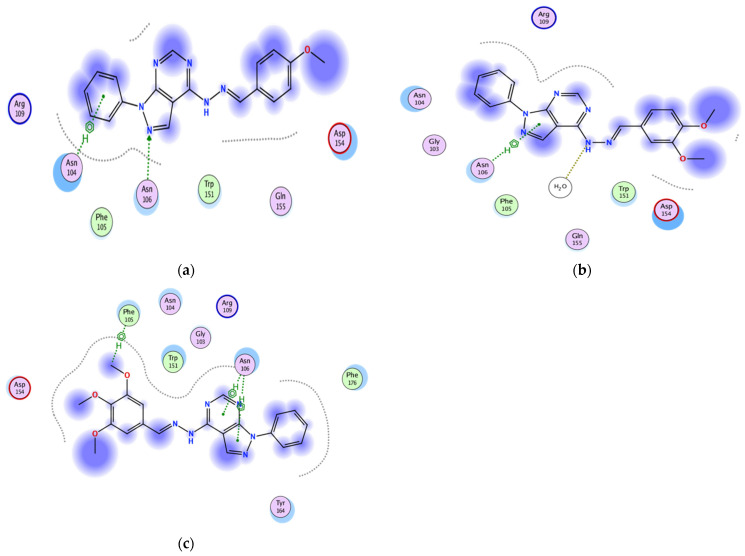
Two-dimensional binding mode of the test compounds (**a**) **6**, (**b**) **5**, and (**c**) **7** in the binding site of Bax protein (PDB ID: 6eb6) using MOE software.

In the case of the binding mode of compound **5** (Figure 6a), the results revealed a binding score equal to (4.0295). The X-ray crystal structure revealed that the N-phenyl ring formed an arene–H bonding with Asn104, while its pyrazolo unsubstituted-N accepted a hydrogen bonding from Asn106. Furthermore, hydrophobic interactions were observed with other amino acids.

The binding mode of **6** (Figure 6b) showed a binding score equal to (4.7846). The X-ray crystal structure revealed an arene–H bonding with Asn106, in addition to hydrophobic interactions with several amino acids.

Figure 6c shows the binding mode of **7**, indicating a superior binding score of (−5.1502) relative to **5** and **6** (−4.0295 and 4.7846, respectively). Furthermore, three arene–H bondings between the methoxy and Phe105, the pyrimidine and Asn106, and finally the pyrazole ring and Asn106, in addition to the hydrophobic interactions with several other amino acids. Surprisingly enough, water molecules were not involved in any of the bonding between the protein and any of the tested compounds, despite its involvement in the binding between the protein and the reported ligand.

#### 2.3.5. Docking of the Test Compounds (**5**–**7**) in the Binding Site of Bcl-2 Protein

A docking study was carried out using the enzyme parameters obtained from the Protein Data Bank of the structure of Bcl-2, having the PDB ID: 5whh [19] for the synthesized compounds (**5**–**7**) showing the following results.

**Figure 7:**
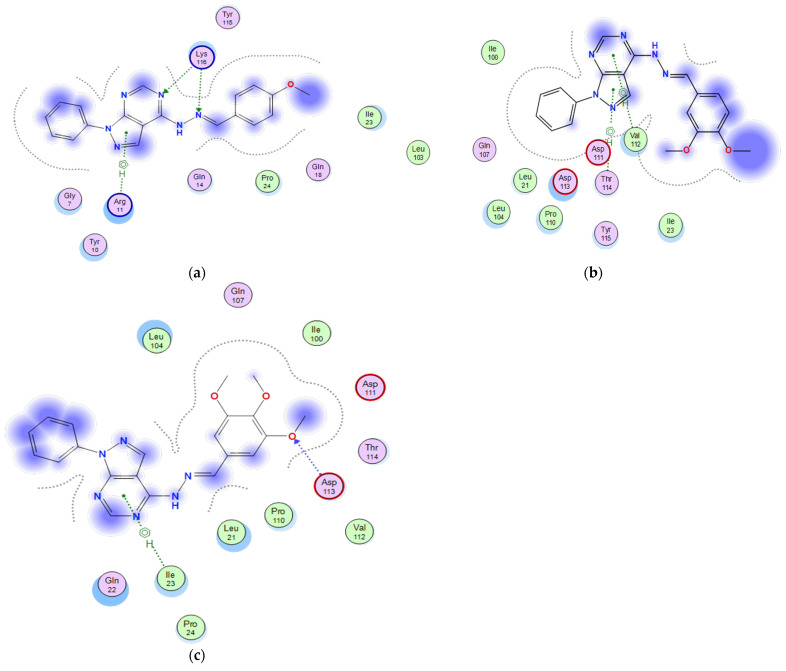
Two-dimensional binding mode of the test compounds (**a**) **6**, (**b**) **5**, and (**c**) **7** in the binding site of Bcl-2 protein (PDB ID: 5whh) using MOE software.

In the case of the binding mode of compound **5** (Figure 7a), the results revealed a binding score equal to (−5.9126). The X-ray crystal structure revealed that the amino acid Lys116 donates two hydrogen bondings, one to the hydrazono-N and the other to the pyrimidine-N, while the pyrazolo nucleus forms an arene–H bonding with Arg11, in addition to various hydrophobic interactions with other amino acids.

The binding mode of **6** (Figure 7b) showed a binding score equal to (−5.6649). The X-ray crystal structure revealed that the pyrazolo nucleus forms two arene–H bondings with Val112 and Thr 114, in addition to hydrophobic interactions with several amino acids.

Figure 7c shows the binding mode of **7**, indicating a superior binding score of (−6.5306) relative to **5** and **6** (−5.9126 and −5.6649, respectively). Furthermore, the pyrazolo ring accepts a hydrogen bonding from Asp113 and an arene–H bonding between the pyrimidine ring and Ile23, in addition to the hydrophobic interactions with several other amino acids.

#### 2.3.6. Docking of the Test Compounds (**5**–**7**) in the Binding Site of P21 Protein

The docking study was carried out using the enzyme parameters obtained from the Protein Data Bank of the structure of P21, having the PDB ID: 6nzv [20].

In a trial to predict the binding modes for the test compounds (**5**–**7**), a docking simulation has been performed, and the following results were obtained.

**Figure 8:**
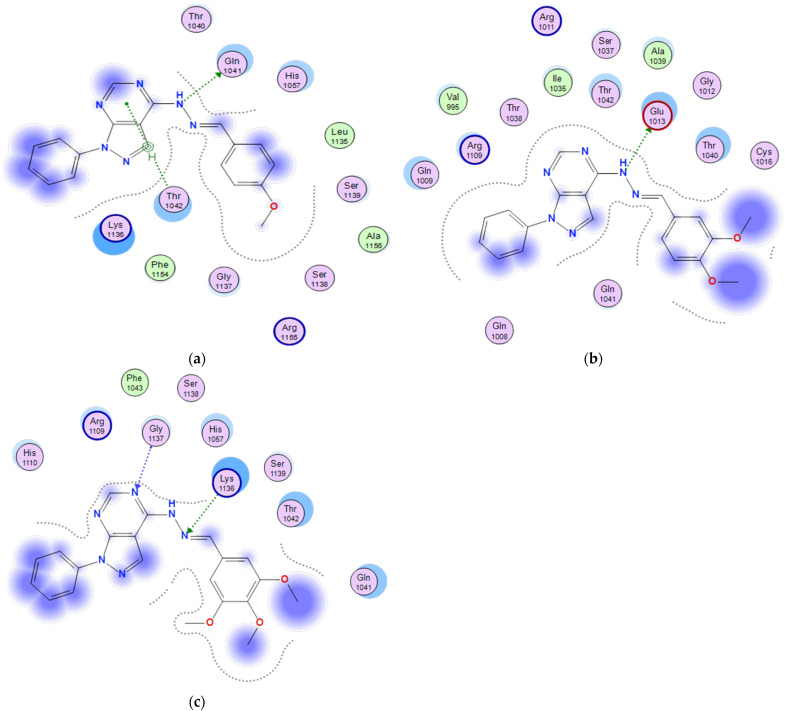
Two-dimensional binding mode of the test compounds (**a**) **6**, (**b**) **5**, and (**c**) **7** in the binding site of P21 protein (PDB ID: 6nzv) using MOE software.

In the case of the binding mode of compound **5** (Figure 8a), the results revealed a binding score equal to (−6.6203). The X-ray crystal structure revealed that the pyrimidine ring formed an arene–H bonding with Thr1042. Furthermore, the Gln1041 formed an arene–H bonding to the hydrazono-NH, in addition to various hydrophobic interactions with other amino acids.

The binding mode of **6** (Figure 8b) showed a binding score equal to (−6.4258). The X-ray crystal structure revealed a hydrogen bonding between Glu1013 and hydrazono-NH, in addition to hydrophobic interactions with several amino acids.

Figure 8c shows the binding mode of **7**, indicating a binding score of (−6.3091). Furthermore, it showed two hydrogen bondings, one between the hydrazono-N and Lys1136 and the other between the pyrimidine-N and Gly1137, in addition to the hydrophobic interactions with several other amino acids.

### 2.4. Cell Viability with MTT

**Figure 9:**
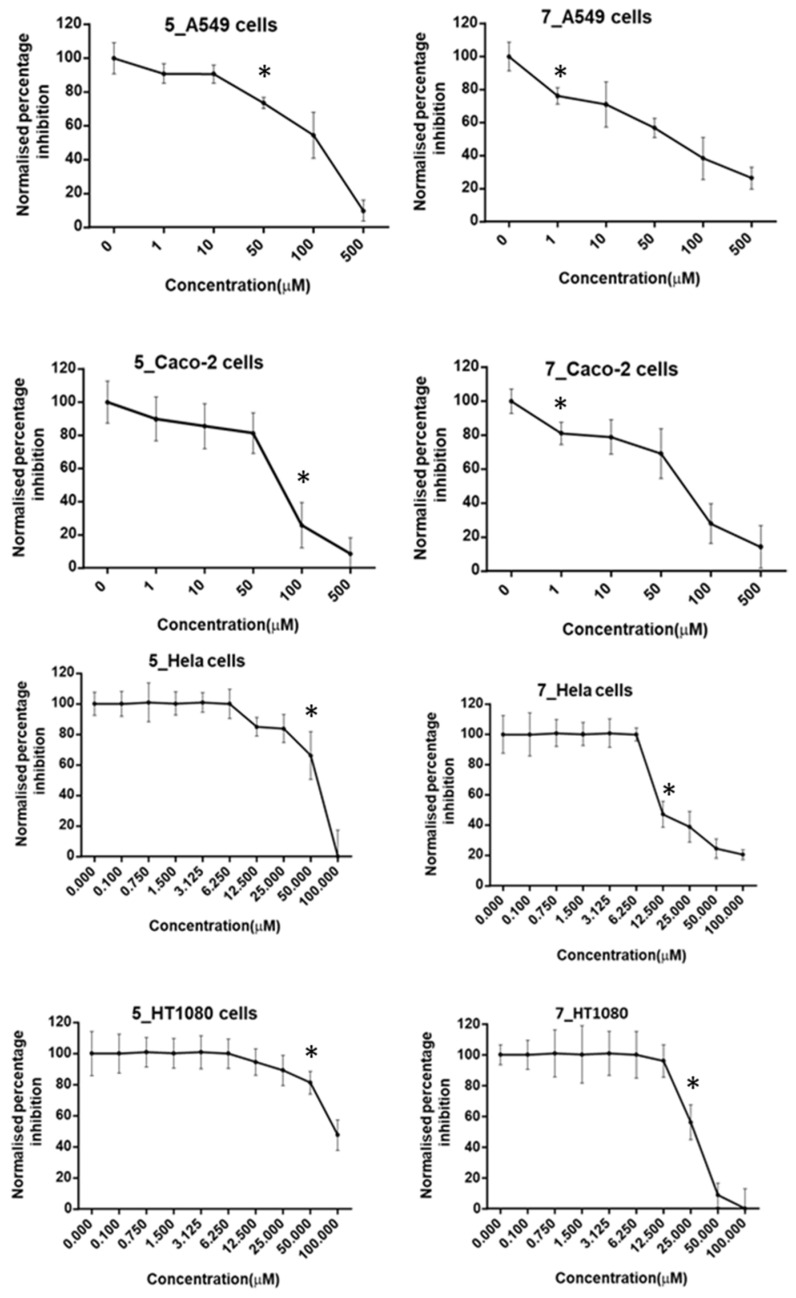
Normalised percentage cell viability inhibition of compounds **5** and **7**, measured by three independent MTT assays in A549 and Caco-2 cell lines and by three independent experiments with crystal violet staining in Hela and HT1080 cell lines. Cells were treated with different concentrations (from 1 and up to 500 µM) of the compounds for 48 h, * = first concentration significant reduction in cell viability in comparison to the control.

A (4,5-dimethylthiazol-2-yl)-2,5-diphenyltetrazolium) MTT assay was used for the determination of the cytotoxic effects of three compounds (**5**, **6**, and **7**) in vitro (Figure 9). Hela, HT1080, Caco-2, and A549 cells were treated with various concentrations of the compounds employed in the current study (0, 1, 10, 50, 100, 500 µM) for 48 h. Compound **6** did not show any significant decrease in the cell viability measured in the tested cell lines (data not shown). However, compound **5** showed anticancer activity against HT1080, Hela, Caco-2, and A549 cells with IC_50_ values of 96.25, 74.8, 76.92, and 148 µM, respectively, and with significant reduction in cell viability upon treatment with lower concentrations than the IC_50_ (50, 50, 100, and 50 µM, respectively).

Compound **7** showed anticancer activity against the same cells with IC_50_ values of 43.75, 17.50, 73.08, and 68.75 µM, respectively, with significant reduction in cell viability upon treatment with lower concentrations than the IC_50_ (25, 12.5, 1, and 1 µM, respectively).

For the first application of the in silico results to biological system, two main proteins cleaved by caspase-3 were examined for their expression in the cell lines treated with compound **7**, as this was the most promising candidate in the studied compounds. Further assessment of the detailed mechanism will be conducted in the future studies.

### 2.5. Apoptosis Assays

**Figure 10:**
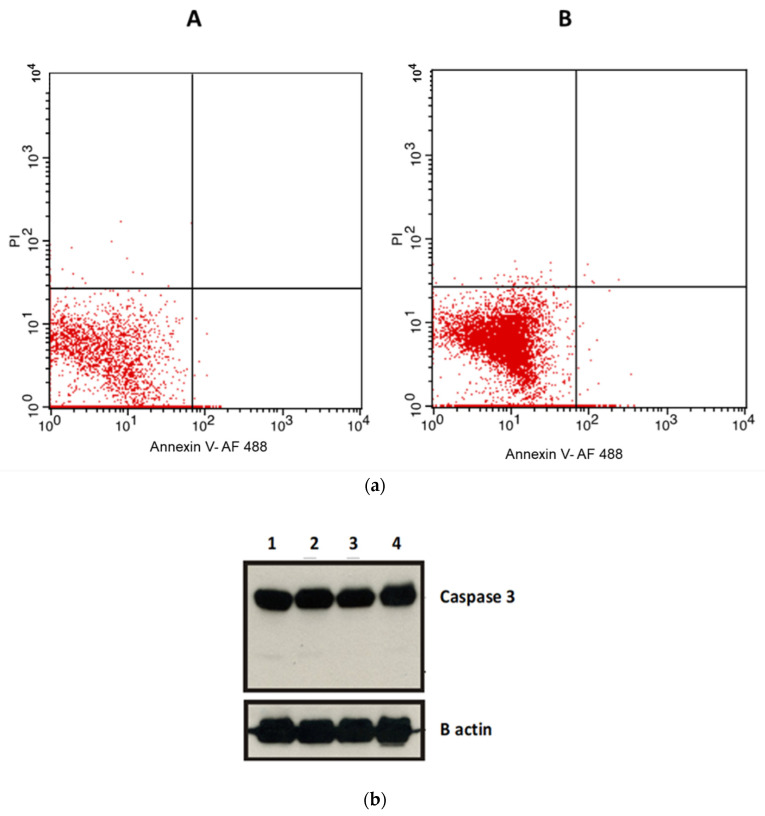

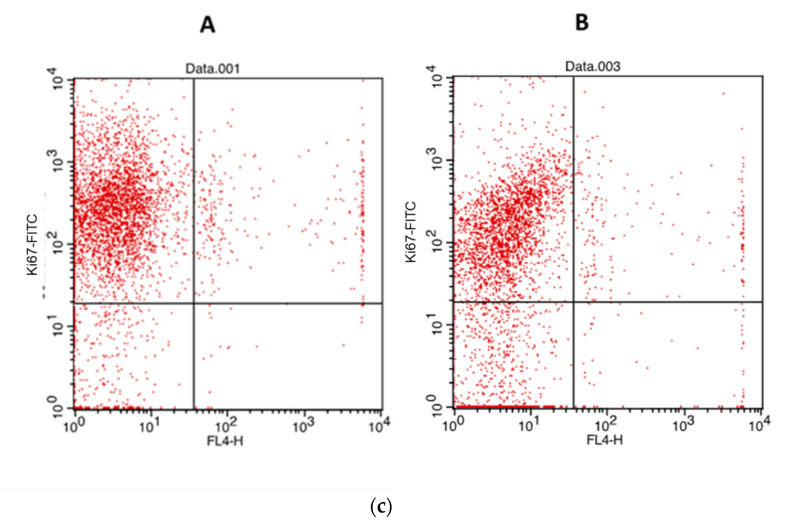
(**a**) Apoptosis analysis in A549 cells following treatment of cells with compound **7** at IC_50_ concentration. Flowcytometry was conducted using Alexa Fluor 488 annexin V conjugate. The percentage gating in the lower left quadrant was (**A**) 97.8 in the control untreated cells and (**B**) 97.58% in compound **7**. (**b**) Western blot analysis showing cleaved caspase-3 protein expression level. The upper panel shows (1) control, (2) compound **5**, (3) compound **6**, and (4) compound **7** at IC_50_ concentration. The lower panel demonstrates B actin as loading control. (**c**) Proliferation analysis (Ki67) in A549 cells following treatment cells with compound **7** at IC_50_ concentration. The analysis was done using flow cytometry and the Ki-67 FITC antibody. (**A**) shows the flow cytometric analysis of the control, whereas (**B**) shows the flow cytometric analysis of compound **7**.

In order to exclude apoptotic effects as a mechanism for the observed anticancer activity of the compounds and according to the in silico results, induction of apoptosis by compound **7** was examined using an annexin V flow cytometry assay (Figure 10a). After incubation of the cells with compound **7** for 24 h, the results showed that there is no significant effect of the treatment with the compound on the viability of A549 cells through apoptosis in comparison to the untreated group. This suggests that the mechanism of action of this anticancer compound is not mediated via apoptosis. These results were further confirmed by western blots. Our results show that there is no significant change in the level of the apoptotic cleaved caspase-3 protein expression compared to the untreated cells (Figure 10b).

### 2.6. Proliferation Assay

Ki67 expression was determined using a flow cytometry assay. Ki67 expression was found to decrease significantly following the cells’ treatment using compound **7** at IC_50_ concentration for 48 h (Figure 10c). The percentage of cells labelled using Ki67 antibody in the control cells was 85.61%, whereas those labelled with the same antibody following treatment with compound **7** was 68.06%. We demonstrated that Ki67 expression was significantly reduced in the cells treated with compound **7**.

## 3. Discussion

Protein kinases have a fundamental role in cell signal transduction cascades that possess several cellular activities (36). Recently, small molecules of protein kinase inhibitor have been broadly tested for antineoplastic properties, such as midazo [1, 5-a] pyrazines, benzotriazines, indoles, and pyrazolopyrimidines. Numerous pyrazolopyrimidines-based compounds have been approved for the treatment of various types of malignancies due to their effectiveness on signal cascades [21].

An MTT assay was used for the determination of the cytotoxic effects of three compounds (**5**, **6**, **7**) in vitro against Hela, HT1080, Caco-2, and A549 cells. Various concentrations of the drugs employed in the current study were inoculated to the cells. According to Brown et al. [5], compound **6** (the 3,4-dimethoxy derivative) was expected to show the highest antitumour activity, where it was introduced in the aforementioned research as a promising protein kinase inhibitor for the treatment of type 2 *diabetes mellitus* T2DM. However, upon testing, it did not show any significant decrease in the cell viability measurements against HT1080 and Hela cells, indicating that despite the fact that it is a protein kinase inhibitor, it does not possess any anticancer activity against the selected cell lines. Nevertheless, it is worth testing further whether it will be able to reduce the measurement of cell viability against other cell lines that might have protein kinase as a part of their survival mechanism, and it will be a part of a future study conducted in our laboratory.

Testing the other two derivatives, compounds **5** (the monomethoxy) and **7** (the trimethoxy), on the other hand, showed promising results against the cell lines used, with compound **7** being the most active. These results indicate that the presence of one methoxy group at the 4-position, as in compound **5**, is preferred for moderate antitumour activity against selected cell lines; however, the addition of a second methoxy group at the 3-position totally abolished the activity. Surprisingly enough, the introduction of a third methoxy group resulted in an enhancement of the activity, as observed in compound **7**, which resulted in the highest antitumour activity. These results indicate that the additional methoxy group is essential for improving the activity.

It was previously confirmed that celecoxib (diaryl-substituted pyrazole structure) electively inhibits cyclo-oxygenase-2 activity (COX-2); COX-2 inhibition may result in apoptosis and a reduction in tumours’ angiogenesis and metastasis [22]. Moreover, celecoxib was found to inhibit the growth of hepatocellular carcinoma cells by activating retinoblastoma protein (pRb) through its hypophosphorylation, thus repressing the DP1/E2F1 complex, and inducing apoptosis through the activation of caspase-3 and caspase-9 [23].

Different molecular mechanisms were reported for cell death, including intrinsic and extrinsic apoptosis, mitochondrial permeability transition (MPT)-driven necrosis, necroptosis, pyroptosis, entotic cell death, lysosome-dependent cell death, autophagy-dependent cell death, immunogenic cell death, cellular senescence, mitotic catastrophe, and other different uncommon mechanisms [24,25].

In order to determine the mechanism of action of compounds **5** and **7**, apoptotic assays were conducted using an annexin flow cytometry assay. Contrary to previous literature [26,27], our results show that there is no significant effect of the treatment of experimental compounds on the viability of cells with regard to the apoptotic level against A549 cells compared to the control untreated group. This suggests that the mechanism of action of this anticancer compound is not mediated via apoptosis. These results were further confirmed using the western blot technique. Our results show that there is no significant change in the level of caspase-3 expression compared to the control untreated cells. On the other hand, a new pyrazolopyrimidine analogue multiple tyrosine kinase inhibitor (CLM3) performed both antiproliferative and proapoptotic properties via a mechanism mediated by the suppression of phosphorylation of extracellular signal regulated kinases ERK1/2 and protein kinase B Akt, respectively [28].

Ki67 expression level was found to be decreased significantly following cells’ treatment using compound **7**. Ki67 is a nuclear protein that is associated with cellular proliferation. It was reported before that Ki67 expression was decreased following chemotherapy. The expression of Ki67 is strongly associated with tumour proliferation and growth. Therefore, Ki67 is widely used as a biomarker for cancer proliferation. The Ki67 antigen encodes two protein isoforms. Expression of Ki67 is low in the G1 phase and is increased during the S and G2 phases. It reaches the maximum expression in the M phase. A significant reduction in Ki67 expression levels occurs in the later phases of mitosis [29,30,31]. A prior study indicated that pyrazolopyrimidine, PP2, diminished RET/PTC1-mediated mitogen-activated protein (MAP) kinases signalling. Moreover, PP2 controlled the proliferation and the invasive phenotype of human thyroid carcinoma cells [32].

Comparing the docking studies results on Ki67 (an indicator of cellular proliferation) with those of caspase-3 (an indicator of apoptosis) revealed that a much better binding of compound **7** was demonstrated towards Ki67, where, in addition to one weak arene–H bonding, the compound formed two hydrogen bonds with Ki67. On the other hand, compound **7** showed only one arene–H bond with caspase-3. Reviewing the above, it was found to be highly consistent with the experimentally obtained results, which indicates that compound **7** inhibits cell growth through inhibition of cellular proliferation rather than apoptosis.

## 4. Materials and Methods

### 4.1. Chemistry

The synthetic procedure of the target compounds was done according to reported procedures [5,10,33], resulting in the new unreported compounds **5** and **7,** and is available in the Supplementary Materials.

*2-(4-Methoxybenzylidene)-1-(1-phenyl-1H-pyrazolo[3,4-d]pyrimidin-4-yl)hydrazine* (**5**), White crystals (65 mg, 86%), m.p: 183–185 °C. IR (KBr, cm^−1^): 3427 (NH), 2885, 3039, 3111 (Ar-H), and 1675, 1597 and 1543 (C=N and Ar-C=C), 1253, 1171 (C-O). ^1^H-NMR (400 MHz, DMSO-*d*_6_, δ ppm): 4.05–4.07 (broad s, 3H, OCH_3_), 7.24 (singlet, 1H, benzyli dene-CH=N), 7.37–7.49 (m, 4H, Ar-benzylidene-C3,5-H and pyrazolophenyl-C2,6-H), 7.55–7.58 (dist t, 3H, pyrazolophenyl-C3,4,5-H), 8.03–8.05 (d, 2H, Ar-benzylidene-C2,6-H), 8.21 (s, 1H, pyrazolopyrimidine-C3-H), 8.35 (s, 1H, pyrazolopyrimidine-C6-H), 12.56 (s, 1H, NH, D_2_O exchangeable). ^13^C-NMR (100 MHz, δ_ppm): 56 (OCH_3_), 108, 114, 122, 123, 124, 127, 130, 132, 136, 138, 139, 149, 152, 157 (Ar-C). Anal. Calcd for C_19_H_16_N_6_O (344.37): C, 66.27; H, 4.68; N, 24.40. Found: C, 66.08; H, 4.56; N, 24.27.

*2-(3,4,5-Trimethoxybenzylidene)-1-(1-phenyl-1H-pyrazolo[3,4-d]pyrimidin4yl)hydrazine* (**7**), White crystals (80 mg, 90%), m.p: 196–198 °C. IR (KBr, cm^−1^): 3427 (NH), 2938, 2835 (Ar-H), and 1665, 1599 (C=N and Ar-C=C), 1281, 1126 (C-O). ^1^H-NMR (400 MHz, DMSO-*d*_6_, δ ppm): 4.39 (broad s, 9H, OCH_3_), 7.26 (singlet, 1H, benzylidene-CH=N), 7.37–7.41 (t, *J* = 8 Hz, 1H, pyra zolophenyl-C4-H), 7.57–7.61 (t, *J* = 8 Hz, 2H, pyrazolophenyl-C3,5-H), 7.69–7.73 and 7.94–7.98 (d, *J* = 16 Hz, 2H, Ar-benzylidene-C2,6-H), 8.21-8.26 (d, *J* = 20 Hz, 2H, pyrazolophenyl-C2,6-H), 8.67 (s, 1H, pyrazolopyrimidine-C3-H), 8.92 (s, 1H, pyrazolopyrimidine-C6-H), 11.01 (s, 1H, NH, D_2_O exchangeable). ^13^C-NMR (100 MHz, δ ppm): 56.3, 56.6, 60 (3 “OCH_3_”), 106, 120, 121.3, 121.6, 127, 129, 130, 132, 134, 139, 140, 144.2, 144.3, 153.4, 153.6, 153.8, 154, 156 (Ar-C). Anal. Calcd for C_21_H_20_N_6_O_3_ (404.42): C, 62.37; H, 4.98; N, 20.78. Found: C, 62.21; H, 4.96; N, 21.07.

### 4.2. In Vivo Toxicity Evaluation

Male albino rats (150 g average weight) were purchased from the animal house of the Institute of Graduate Studies and Research, Alexandria University, Egypt. The animals were housed under standardized environmental conditions, fed with a standard diet and water, and left to acclimate to the environment for one week prior to inclusion in the experiment. All of the animal experiments were conducted in accordance with the guidelines for the care and use of laboratory animals of the National Institutes of Health (NIH 1985). The experimental procedures employed were ethically reviewed and approved by the Institutional Animal Care and Use Committee (IACUC) of Alexandria University (Ethical approval number of the study: AU14-200922-1-8A) and “declaration of Helsinki”. Four groups of rats were administered 20 mg/kg/day of compounds **5**, **6**, and **7** orally twice weekly for 6 weeks. Liver enzymes and other liver biomarkers together with creatinine and urea were measured (Table 1). All of the results of the former parameters revealed that compounds **5**, **6**, and **7** are safe compounds comparable to the healthy control group.

#### Assessment of Toxicity Parameters

The collected blood was allowed to clot, the serum was separated at 2000× *g*, and the biochemical markers of hepatic damage, including serum ALT, AST [33], ALP [34], albumin [35], total bilirubin [36], TG [37], urea [38], creatinine [39], and total cholesterol [40], were estimated according to previously reported methods. These parameters were determined using available commercial kits (ARE) following the instructions of the manufacturer.

### 4.3. Docking Study

Computer-assisted simulation docking experiments were carried out under an MMFF94X force field using Molecular Operating Environment (MOEDock 2015) software, Chemical Computing Group, Montreal, QC, Canada.

#### Docking Protocol

The coordinates from the X-ray crystal structure of Ki67, caspase-3, P53, Bax, Bcl-2, and P21 used in this simulation were obtained from the Protein Data Bank (PDB ID: 2aff, 6bdv, 5mct, 6eb6, 5whh, and 6nzv, respectively), where the active site is bound to the appropriate ligand. The ligand molecules were constructed using the builder molecule and were energy minimized. The active sites of the proteins were generated using the MOE-Alpha Site Finder, and then ligands were docked within this active site using the MOE Dock. The lowest energy conformation was selected, and the ligand interactions (hydrogen bonding, arene–H, arene–arene interactions together with other hydrophobic interactions) with the proteins were recorded.

### 4.4. Cell Culture

Human fibrosarcoma (HT1080), cervical cancer (Hela), colorectal cancer (Caco-2), and human lung cancer cell lines (A549) were purchased from American Type Culture Collection (ECACC, Salisbury, UK). Cervical cancer cells (Hela) and fibrosarcoma cells (HT-1080) were maintained in a RPMI medium supplemented with 10% foetal bovine serum (FBS) and were expanded at 37 °C and 5% CO_2_. Caco-2 and A549 cells were grown in Dulbecco’s modified eagle medium (DMEM)-high glucose (biowest, Riverside, MO, USA) enriched with (10% *v*/*v*) (foetal bovine serum) FBS (biowest, Riverside, MO, USA) and antibiotics (100 U/mL penicillin, 100 μg mL streptomycin) (Lonza, Basel, Switzerland). Cells were maintained at 37 °C in an incubator with 5% CO_2_ atmosphere. Part of the experiments were carried out in CERRMA (Center of Excellence for Research in Regenerative Medicine and its Applications), Faculty of Medicine, Alexandria University.

### 4.5. Experimental Design

Hela, Caco-2, HT1080, and A549 cancer cell lines were screened for their integrity and to ensure that they were free from contamination before starting the study and then cultured as previously mentioned [41,42,43,44].

### 4.6. Cell Viability Assays

The cell viability of A549 and Caco-2 cells was measured using MTT assays. Cells were incubated for 48 h in triplicates with the compounds of interest. The MTT assay shows the effect of the drug on the viability and proliferation of different cancer cell lines. Cells were seeded in 96-well plates at a density of 5 × 10^3^ cells/well and were maintained in complete media. After adherence for 48 h, cells were treated with different concentrations of newly synthesized compounds ranging from 10 to 500 μM. The plates were incubated at 5% CO_2_ and 37 °C. After 48 h, the medium was discarded, and cells were incubated with 100 μL MTT (Serva, Heidelberg, Germany) solution (0.5 mg/mL) for 3 h at 5% CO_2_ and 37 °C. Finally, the reagent was discarded and replaced with 100 μL DMSO. The optical density was measured at a wavelength of 570 nm (Tecan, Infinite F50, Magellan reader control and data analysis software, Mennedov, Switzerland). The percentage cell viability data were obtained by normalization of the optical density (OD) values for all groups to the OD of the control group.

Cervical Hela and HT-1080 cells (2 × 10^4^ per well) were seeded in 96-well tissue culture plates in 100 μL cell culture medium. Cells were incubated for 48 h in triplicates with the compounds of interest. The cytotoxic effect was assessed by crystal violet staining and measured at 595 nm. To normalize cell viability values each plate included a triplicate of cells treated with DMSO, the vehicle of the compound to calculate 100% viable cells. At the same time, a triplicate of cells was simulated with a cytotoxic combination (200 ng/mL TNF, 200 ng/mL CD95L, 200 ng/mL TRAIL, 25 µg/mL CHX, 1% (*w*/*v*) sodium azide) to elucidate maximal cell death, and thus 0% viability. All other viability values were normalized depending on the averages of these triplicates and evaluated by the Graph Pad Prism 5 software (La Jolla, CA, USA).

### 4.7. Apoptosis Assays

For evaluating cell apoptosis, cells were plated in 6-well plates at a density of 2 × 10^4^ cells per well. After incubation for 24 h, 70% confluency is reached. Cells were then labelled using the FITC Annexin V apoptosis detection kit I (BD Pharminogen, San Diego, CA, USA) according to manufacturer’s instructions (Cell Signaling Technology; Flow Cytometry, Methanol Permeabilization Protocol) and analysed by using BD FACS Calibur flow cytometry and Cell Quest™ software.

### 4.8. Cell Proliferation Assay

For evaluating the proliferation status of cells, cells were plated in 6-well plates at a density of 2 × 10^4^ cells per well for the cell proliferation assay. After incubation for 24 h, 70% confluency is reached. Cells treated with IC_50_ concentration of the tested compound for 48 h were labelled using the Ki67 Proliferation Kit (D3B5, Rabbit mAb Alexa Fluor^®^ 488 Conjugate, Cell Signaling Technology) according to manufacturer’s instructions (Cell Signaling Technology; Flow Cytometry, Methanol Permeabilization Protocol) and analysed by using BD FACS Calibur flow cytometry and Cell Quest™ software.

### 4.9. Investigation of Caspase-3 Protein Expression Levels Involved in the Anticancer Effect

A western blot assay was carried out to examine cleaved caspase-3 protein expression levels following the different treatments with different concentrations of the compound that were employed in the current study. Cells (5 × 10^5^ cells/well) were seeded in 12-well plates and were induced the next day with the compounds of interest for 48 h. The cells were collected and centrifuged (2 min, 10,000× *g*). The cells were washed two times with ice-cold PBS, and the cell pellet was suspended in 4× Laemmli sample buffer (8% (*w*/*v*) SDS, 0.1 M dithiothreitol, 40% (*v*/*v*) glycerol, 0.2 M Tris, pH 8.0) supplemented with a phosphatase inhibitor cocktail II (Sigma) by sonification (20 pulses) and heating for 5 min at 95 °C. After removal of the remaining insoluble debris by centrifugation (2 min, 10,000× *g*) 8 µL of the lysate were applied to SDS–PAGE. The isolated proteins were blotted from the gel to a nitrocellulose. The proteins of interest were detected with a suitable primary antibody (Anti-Caspase-3, 17,19 kD, Rabbit polyclonal ≠ 9661, from Cell Signaling Technology, Beverly, MA, USA) and horseradish peroxidase conjugated secondary antibodies (Dako, Glostrup, Denmark and Cell Signaling Technology, Beverly, MA, USA).

#### Statistics

The in vitro data are representative of at least three independent experiments and shown as mean ± standard deviation (SD). Statistical significance among multiple groups was tested with the Anova post hoc tukey test. A *p* value of less than 0.05 is considered to be statistically significant for all results.

## 5. Conclusions

The target of the present study was to synthesize and evaluate the antineoplastic activities of some new pyrazolo[3,4-d]pyrimidines. The obtained results clearly revealed that compounds **5** and **7** are promising antineoplastic agents against four cancer cell lines investigated in the current study and thus represent a fruitful matrix that deserves further investigation and derivatization.

## Figures and Tables

**Table 1:** Measurement of the change in the hepatic and renal biochemical parameters in rats treated with the investigated compounds.

	Control Group	5	6	7
Cholesterol (mg/dL)	70 ± 3.1	71 ± 4.8	69 ± 7.8	71 ± 5.8
Triglyceride (mg/dL)	74 ± 5.1	72 ± 8.6	75 ± 6.5	77 ± 7.5
Bilirubin (mg/dL)	0.12 ± 0.13	0.11 ± 0.12	0.12 ± 0.12	0.11± 0.32
Albumin (mg/dL)	4.5 ± 0.12	4.4 ± 0.37	4.6 ± 1.8	4.6 ±1.1
ALT (U/L)	44 ± 4.1	43 ± 5.2	44 ± 5.7	42 ± 7.2
AST (U/L)	103 ± 4.6	102 ± 8.7	100 ± 9.8	103 ± 8.8
ALP (U/L)	1.92 ± 0.47	2.1 ± 0.52	2 ± 0.12	1.8 ± 0.88
Urea (U/L)	5.8 ± 0.23	5.7 ± 0.98	5.8 ± 0.80	5.88 ± 0.90
Creatinine (mg/dL)	28 ± 2.5	27 ± 4.2	29 ± 3.86	28 ± 3.86

## Data Availability

All results and data are given in the manuscript and its supplements. Further details are available from the corresponding author. The data presented in this study are available on request from the corresponding author.

## Supplementary Materials

The following are available online, a description of all reagents, solvents and instrumentation used in the chemistry part is supplied together with the general procedure for the synthesis of the target compounds. In addition the yield and m.p. of the reported compound (**6**) are given.

## Sample Availability

Samples are limited, but we have protocols and can advise how to generate them.
