# Supplementary material for: Design, Synthesis, and Anticancer Screening for Repurposed Pyrazolo[3,4-d]pyrimidine Derivatives on Four Mammalian Cancer Cell Lines
Source: Molecules. 2021 May 16;26(10):2961. doi: 10.3390/molecules26102961 (PMC8156061; doi:10.3390/molecules26102961)
Supplement: Supplementary file 1 [file molecules-26-02961-s001.zip › molecules-1196117-supplementary.pdf]

Supplementary data

Chemistry

All reagents and solvents were purchased from commercial suppliers and were dried and purified when necessary by standard techniques. Melting points were determined in open glass capillaries using Stuart capillary melting point apparatus (Stuart scientific Stone, Staffordshire, UK) and are uncorrected. Infrared (IR) spectra were recorded on Perkin-Elmer 1430 infrared spectrophotometer (Perkin Elmer, Beaconsfield, UK) and measured by ν' cm^{-1} scale using KBr cell. NMR spectra were scanned on Bruker-400 MHz spectrometer using tetramethylsilane (TMS) as internal standard and DMSO-d6 as solvent (chemical shifts are given in δ ppm). Splitting patterns were designated as follows: s: singlet; d: doublet; t: triplet; m: multiplet; dist: distorted. Microanalyses (for purity) were performed at the regional Center for Mycology and Biotechnology, Al-Azhar University and the found values were within ± 0.4% of theoretical values. Follow up of the reactions and checking the purity of the compounds was made by thin layer chromatography (TLC) on silica gel-precoated aluminum sheets (Type 60 GF254; Merck; Germany) and the spots were detected by exposure to UV lamp at λ 254 nm for few seconds.

General procedure for the preparation of 2-arylidene-1-(1-phenyl-1H-pyrazolo[3,4-d]pyrimidin-4- yl)hydrazine (5-7)

Equimolar amounts of 1-(1-phenyl-*1H*-pyrazolo[3,4-d]pyrimidin-4-yl)hydrazine **(4)** (0.22 mmol, 50 mg) and the appropriate aromatic aldehyde in absolute ethanol (5 ml) were heated under reflux in the presence of piperidine (2 drops) for 4 hours. The reaction mixture was allowed to cool to room temperature depositing an off white solid. The resulting solid was filtered and washed with ethanol, dried and crystallized from ethanol.

2-(3,4-dimethoxybenzylidene)-1-(1-phenyl-1H-pyrazolo[3,4-d]pyrimidin-4-yl)hydrazine (6)[5] White crystals (60 mg, 73 %), m.p:189-192°C.
